# Bone marrow-derived cells in the population of spinal microglia after peripheral nerve injury

**DOI:** 10.1038/srep23701

**Published:** 2016-03-23

**Authors:** Ryoichi Tashima, Satsuki Mikuriya, Daisuke Tomiyama, Miho Shiratori-Hayashi, Tomohiro Yamashita, Yuta Kohro, Hidetoshi Tozaki-Saitoh, Kazuhide Inoue, Makoto Tsuda

**Affiliations:** 1 Department of Life Innovation, Graduate School of Pharmaceutical Sciences, Kyushu University, Fukuoka 812-8582, Japan; 2 Department of Molecular and System Pharmacology, Graduate School of Pharmaceutical Sciences, Kyushu University, Fukuoka 812-8582, Japan

## Abstract

Accumulating evidence indicates that peripheral nerve injury (PNI) activates spinal microglia that are necessary for neuropathic pain. Recent studies using bone marrow (BM) chimeric mice have reported that after PNI, circulating BM-derived cells infiltrate into the spinal cord and differentiate into microglia-like cells. This raises the possibility that the population of spinal microglia after PNI may be heterogeneous. However, the infiltration of BM cells in the spinal cord remains controversial because of experimental adverse effects of strong irradiation used for generating BM chimeric mice. In this study, we evaluated the PNI-induced spinal infiltration of BM-derived cells not only by irradiation-induced myeloablation with various conditioning regimens, but also by parabiosis and mice with genetically labelled microglia, models without irradiation and BM transplantation. Results obtained from these independent approaches provide compelling evidence indicating little contribution of circulating BM-derived cells to the population of spinal microglia after PNI.

Neuropathic pain occurring after peripheral nerve injury (PNI) is a highly debilitating chronic pain state and is often resistant to all treatments currently available, including non-steroidal anti-inflammatory drugs and opioids^1^,^2^. Therefore, unravelling the molecular and cellular mechanisms for the development and maintenance of neuropathic pain is necessary for the discovery of new treatments for neuropathic pain. A growing body of evidence from diverse animal models of neuropathic pain indicates that spinal cord microglia play a pivotal role in the development and maintenance of PNI-induced pain hypersensitivity^2^,^3^,^4^,^5^,^6^,^7^,^8^. Thus, microglia have received much attention as key players for discovering new mechanisms of neuropathic pain and as potential therapeutic targets^7^,^9^,^10^,^11^.

Microglia are known as tissue-resident macrophages in the central nervous system (CNS) and constitute 5–20% of the total cells in the adult CNS^12^,^13^,^14^. Recent evidence indicates that the origin of microglia are primitive macrophages in the yolk sac^11^,^15^,^16^. Fate mapping studies have revealed that microglia arise from early yolk sac-derived precursors that leave the yolk sac, migrating to the neuroectoderm via the primitive bloodstream^17^. These precursors have erythromyeloid potential^18^. Interestingly, microglial generation is independent of the transcription factor Myb^19^, which is essential for bone marrow (BM)-derived macrophages^20^,^21^. Therefore, it is assumed that microglia might remain throughout life and be maintained by self-renewal in the healthy adult CNS with little contribution from BM-derived monocytes/macrophages^22^.

Under pathological conditions, microglia become activated and more numerous (referred to as microgliosis). Following PNI, spinal microglia are positive to proliferation markers^8^,^23^,^24^,^25^,^26^,^27^. The proliferation activity of microglia peaks around 2 days after PNI and then declines to basal levels^8^,^23^,^25^,^27^, which results in an increase in their number by 2- to 4-fold^3^,^28^,^29^,^30^. These findings have suggested an acute, local expansion of spinal microglia after PNI. However, it has been reported that a considerable percentage (about 30–40%) of spinal microglia after PNI might be BM-derived cells that infiltrate into the spinal dorsal horn parenchyma presumably through peripheral blood circulation^31^,^32^,^33^,^34^. Interestingly, these infiltrated BM-derived cells have proliferative activity following PNI, express ionized calcium-binding adapter molecule-1 (Iba1, a marker of microglia/macrophages) and show a microglia-like morphology^31^. Moreover, recent transcriptome studies have shown that resident microglia have a distinct gene expression profile as compared with circulating monocytes or macrophages derived from BM^35^,^36^,^37^. Thus, these findings raise the possibility that the population of spinal cord microglia after PNI may be phenotypically and functionally heterogeneous. This is intriguing in terms of advancing our understanding on the mechanisms involved in neuropathic pain and also their clinical implications. However, one limitation of these studies is that all were investigated in chimeric mice receiving lethal whole-body irradiation with a high dose coupled with BM transplantation. This strong irradiation has been reported to produce multiple toxic side effects including an induction of chemoattractants and a disruption of the blood brain barrier (BBB)^38^,^39^,^40^,^41^ which might allow the infiltration of circulating blood cells into the spinal cord. Thus, the extent at which BM-derived monocytes/macrophages recruit in the spinal cord after PNI remains an important unresolved question.

In this study, we evaluated the infiltration of BM-derived cells into the spinal cord after PNI using chimeric mice generated using two different approaches: one was myeloablation by whole-body irradiation with various conditioning regimens to reduce undesired side effects, and the other was parabiosis in which two mice were surgically joined, a model without irradiation and transplantation. We also used *Cx3cr1*^GFP/+^ mice in which all microglia are visualised by replacing the *Cx3cr1* gene with the *Egfp* gene^42^. By using *Cx3cr1*^GFP/+^ mice, microglia and peripheral monocytes/macrophages are able to be distinguished by the level of GFP expression^43^,^44^. Our results all show that BM-derived cells do not contribute to the population of spinal microglia after PNI. We propose that PNI-induced spinal microgliosis may not be a consequence of recruitment of BM-derived cells.

## Results

**Figure 1:**
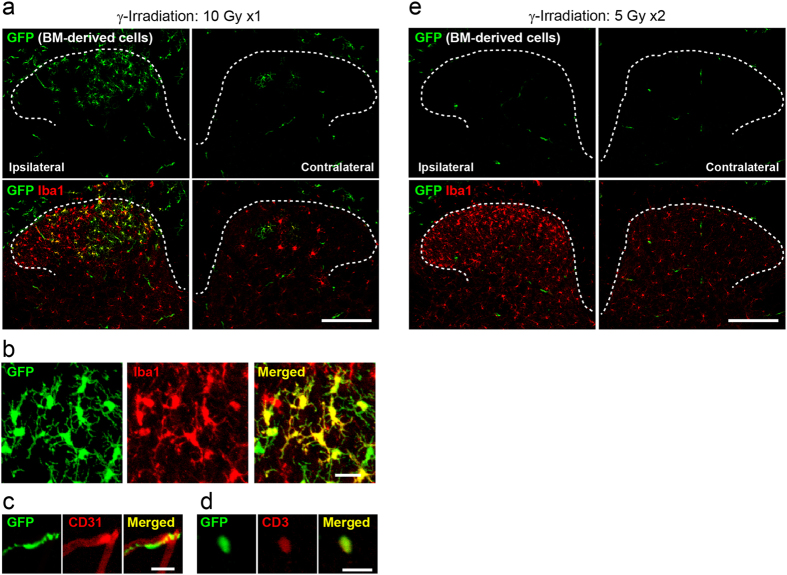
Bone marrow-derived cells in the spinal dorsal horn in γ-irradiated mice with BM transplantation after PNI. (**a**) GFP^+^ bone marrow (BM)-derived cells in the spinal dorsal horn 2 weeks after PNI. C57BL/6 mice were irradiated with a single dose of 10 Gy and transplanted with BM cells from CAG-EGFP mice. GFP and Iba1 are shown in green and red, respectively. Scale bar, 200 μm. (**b–d**) High magnification of GFP^+^ cells in the ipsilateral dorsal horn stained for Iba1, a marker for microglia (**b**), the endothelial cell marker CD31 (**c**), and the T-lymphocyte marker CD3 (**d**). Scale bar, 20 μm. (**e**) GFP^+^ BM-derived cells in the spinal dorsal horn 2 weeks after PNI in BM chimeric mice generated by two doses of 5 Gy irradiation (5 Gy × 2) and GFP^+^ BM transplantation. GFP and Iba1 are shown in green and red, respectively. Scale bar, 200 μm.

To evaluate the infiltration of BM-derived cells into the spinal cord after PNI, we first used BM chimeric mice generated by transplantation of BM cells from transgenic mice ubiquitously expressing green fluorescent protein (GFP) into lethally γ-irradiated adult C57BL/6 mice (single dose of 10 Gy). After 4 weeks, we counted the numbers of GFP^+^ cells in peripheral blood by flow cytometry and confirmed efficient chimerism as demonstrated by the large proportions of circulating blood leukocytes expressing GFP (97.7%). At 8 weeks, the BM chimeric mice were subjected to PNI, and 2 weeks later the recruitment of GFP^+^ BM-derived cells to the spinal dorsal horn was evaluated immunohistochemically, according to previous studies showing that GFP^+^ Iba1^+^ BM-derived cells in the spinal cord are observed 2 weeks after PNI^31^,^32^,^33^,^34^. We observed many GFP^+^ cells in the spinal cord ipsilateral to the PNI compared with the contralateral side where few GFP^+^ cells were present (Fig. 1a). GFP^+^ cells were immunolabelled with Iba1 and displayed a ramified morphology (Fig. 1b). Among BM-derived GFP^+^ cells in the dorsal horn (452 cells from 5 sections), the percentage of Iba1^+^ cells was 83.0% (375 cells), and in this population, ramified morphology was 94.1%. There were also some rod-shaped GFP^+^ cells (9.1% per total GFP^+^ cells) along the vasculature that stained positive for the marker of endothelial cells CD31 (Fig. 1c) and round-shaped cells (4.2% per total GFP^+^ cells) co-labelled with the marker of T cells CD3 (Fig. 1d). These results are comparable to data reported previously^31^.

**Figure 2:**
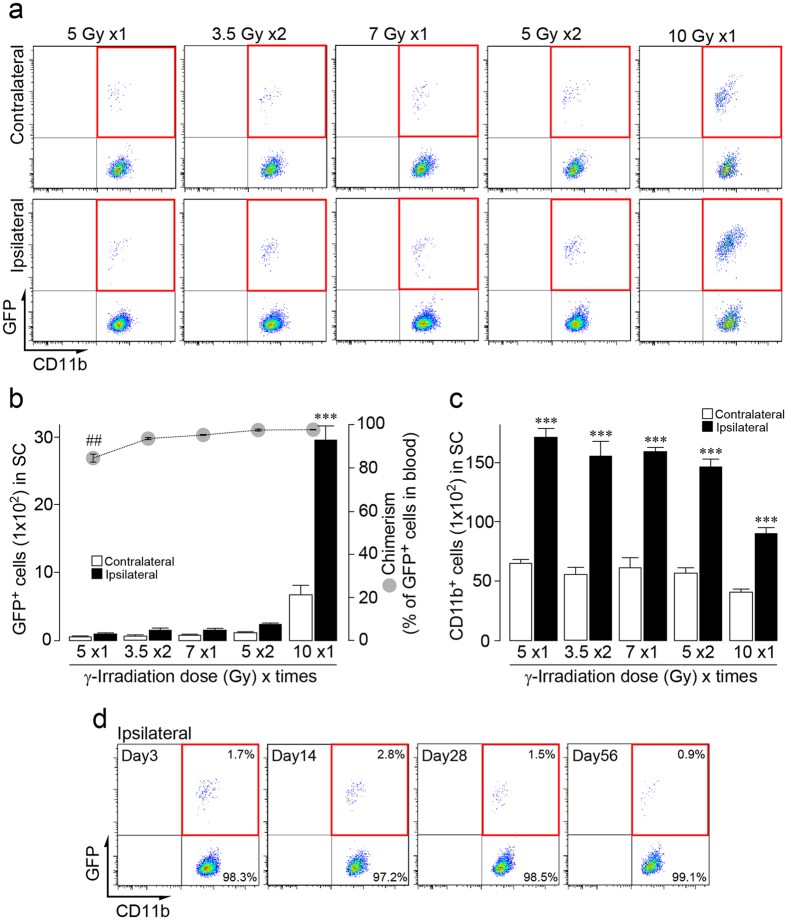
Effects of various doses of γ-irradiation on the infiltration of bone marrow-derived cells in the spinal cord after PNI. (**a**) Representative pseudocoloured scatterplots of CD11b-gated cells in the spinal cord 2 weeks after PNI. Mice were irradiated with various conditioning regimens (1 dose of 5, 7 or 10 Gy; 2 doses of 3.5 or 5 Gy, 3-h interval) and transplanted with GFP^+^ bone marrow (BM) cells. CD11b^+^ cells were delineated by the expression of GFP (Y-axis) and CD11b (X-axis), with the upper-right quadrant (red frame) representing infiltrating CD11b^+^ GFP^+^ BM-derived cells. (**b**) Number of CD11b-gated GFP^+^ BM-derived cells in both sides of the spinal cord 2 weeks after PNI (left Y-axis). n = 4–7, ^***^P < 0.001 vs. contralateral side of group with 10 Gy × 1. Grey circles indicate the percentage of GFP^+^ cells in blood leukocytes (right Y-axis). n = 4–7 mice, ^##^*P* < 0.01 vs. group with 10 Gy × 1. Data are the mean ± SEM. (**c**) Total number of CD11b^+^ microglia in both sides of the spinal cord 2 weeks after PNI. n = 4–7, ^***^P < 0.001 vs. contralateral side of each group. Data are the mean ± SEM. (**d**) Scatterplots of CD11b-gated GFP^+^ BM-derived cells in the ipsilateral spinal cord 3, 14, 28 and 56 days after PNI. The percentage of cells in the upper and lower-right quadrants per total CD11b^+^ cells are shown.

Splitting irradiation in lower doses results in milder toxicity than a single strong dose^41^. Thus, C57BL/6 mice were subjected to split-dose irradiation [two doses of 5 Gy (5 Gy × 2), 3-h interval: total 10 Gy]. We confirmed efficient reconstitution of donor GFP^+^ cells in the blood of split-dose irradiated mice (97.8%), which was identical to lethal single-dose irradiated mice described above. Surprisingly, despite clear reactive microgliosis by PNI (Fig. 1e), we detected substantially less infiltrated GFP^+^ BM-derived cells into the spinal dorsal horn after PNI (Fig. 1e). To quantitatively examine PNI-induced spinal infiltration of BM-derived cells, the number of GFP^+^ cells was assessed by flow-cytometry. Microglia were detected by immunostaining with CD11b, a marker of microglia, in the spinal cord and brain. Indeed, by using *Cx3cr1*^GFP/+^ mice in which all microglia express GFP^42^,^45^, we confirmed by flow-cytometry that almost all CD11b^+^ cells in the spinal cord are positive for GFP (99.8%). In irradiated BM chimeric mice (10 Gy × 1) with PNI, the number of CD11b-gated GFP^+^ cells was increased in the ipsilateral spinal cord, but in split-dose irradiated mice (5 Gy × 2), the cell number dramatically decreased (Fig. 2a,b). We also examined the number of CD11b^+^ GFP^+^ cells in the spinal cord after PNI using BM-chimeric mice with various irradiation doses. We found little contribution from BM-derived cells in the spinal cord after PNI (Fig. 2a,b). However, the percentage of GFP^+^ cells in circulating blood leukocytes was almost identical among the groups with 7 Gy × 1 (95.4%) and 3.5 Gy × 2 (93.7%) (Fig. 2b). Furthermore, PNI-induced microgliosis was similarly induced in the spinal cord: the increase in total CD11b^+^ cells in the ipsilateral spinal cord was on average 2.6-fold (5 Gy × 1), 2.8-fold (3.5 Gy × 2), 2.6-fold (7.5 Gy × 1) and 2.6-fold (5 Gy × 2) over the contralateral side (Fig. 2c). In the group with 10 Gy × 1, the increase in total CD11b^+^ cells was slightly less (2.2-fold), which may be due to a toxic effect of strong irradiation. In addition, we examined the different time points after PNI (day 0, 3, 14, 28 and 56). The number of CD11b-gated GFP^+^ BM-derived cells in the spinal cord was also low at all time-points tested (Fig. 2d). These results showed that the number of CD11b^+^ GFP^+^ cells in the spinal cord of irradiated mice (10 Gy × 1) is exceptionally high and that there is no correlation between peripheral blood reconstitution and GFP^+^ BM-derived cell infiltration into the spinal cord.

**Figure 3:**
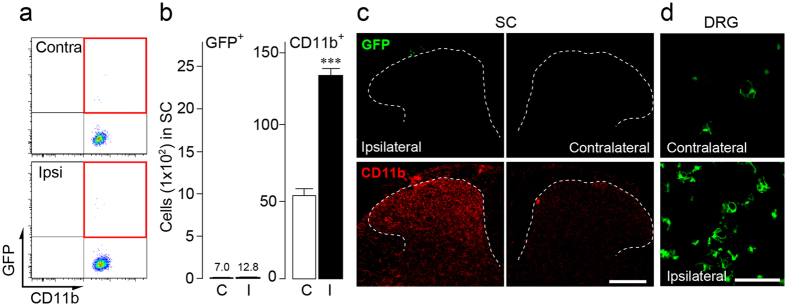
Circulating peripheral blood cells in the spinal cord of parabionts after PNI. (**a**) Representative scatterplots of CD11b-gated cells in the spinal cord of parabiotic mice 2 weeks after PNI. Parabionts (C57BL/6 mice) were surgically joined with CAG-GFP mice. Spinal CD11b^+^ cells in parabionts were delineated by the expression of GFP (Y-axis) and CD11b (X-axis), with the upper-right quadrant (red frame) representing infiltrating CD11b^+^ GFP^+^ cells. (**b**) The number of CD11b-gated GFP^+^ cells and total CD11b^+^ cells in the ipsilateral (I) and contralateral (C) sides of the spinal cord 2 weeks after PNI. n = 5 parabionts, ^***^P < 0.001 vs. contralateral side. Data are the mean ± SEM. (**c,d**) Donor GFP^+^ cells in the spinal dorsal horn (**c**) and DRG (**d**) of parabionts 2 weeks after PNI. CD11b immunofluorescence is shown in red (**c**). Scale bar, 200 μm (**c**) and 100 μm (**d**).

To further examine the contribution of BM-derived cells to the population of spinal microglia after PNI, we used mouse parabiosis, in which parabionts (C57BL/6 mice) had been surgically joined with GFP mice, which in turn shares circulating blood between the two mice. As reported previously^46^, 2 weeks after parabiosis, parabionts had an average of 43.4±1.5% GFP^+^ cells in circulating blood leukocytes (n = 5). The parabionts were subjected to unilateral PNI 4 weeks after establishment of parabiosis. Two weeks later, we assessed the presence of GFP^+^ BM-derived cells in the spinal cord. Flow cytometry revealed that CD11b-gated GFP^+^ cells were almost absent in the spinal dorsal horn of parabionts (Fig. 3a,b), while the PNI-induced microgliosis in the dorsal horn was clearly induced (Fig. 3a,b). The lack of CD11b^+^ GFP^+^ cells and the presence of PNI-induced microgliosis was confirmed by immunohistochemical analyses (Fig. 3c). In addition, CD11b^neg^ CD45^high^ GFP^+^ cells (presumably T-lymphocytes) in the spinal cord was observed after PNI (14.6 ± 1.2% per total CD11b^neg^ CD45^high^ cells), indicating that all types of cells derived from BM do not necessarily infiltrate into the spinal cord after PNI. In the dorsal root ganglia (DRG), GFP^+^ cells were readily observed in the ipsilateral side (Fig. 3d), which is consistent with evidence indicating that a population of macrophages in the DRG are derived from BM cells^47^,^48^. Our data using parabiotic mice provides evidence that circulating peripheral blood cells do not contribute to the spinal microglia population.

**Figure 4:**
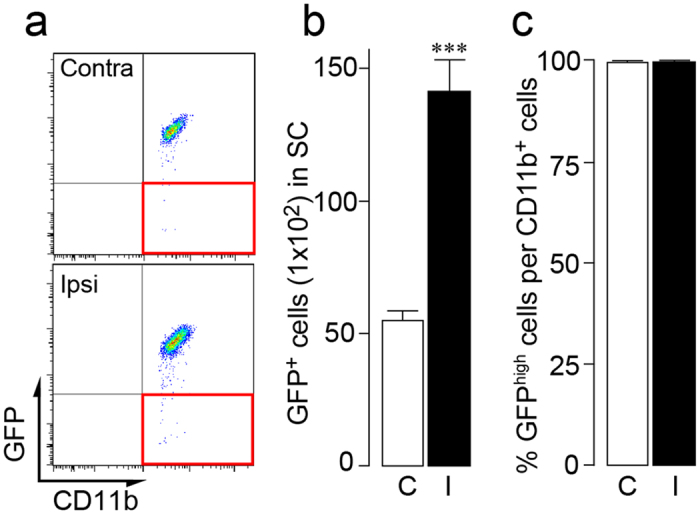
Circulating blood monocytes do not contribute to PNI-induced spinal microgliosis after PNI. (**a**) Representative pseudocoloured scatterplots of CD11b-gated cells in the spinal cord of *Cx3cr1*^GFP/+^ mice 2 weeks after PNI. Spinal CD11b^+^ cells were delineated by the expression of GFP (Y-axis) and CD11b (X-axis), with the lower-right quadrant (red frame) representing infiltrated CD11b^+^ GFP^low/neg^ circulating monocytes. (**b,c**) Total number of GFP^+^ cells (**b**) and the percentage of GFP^high^ per total CD11b^+^ cells (**c**) in the ipsilateral (I) and contralateral (C) spinal cord. n = 4 *Cx3cr1*^GFP/+^ mice, ^***^P < 0.001 vs. contralateral side. Data are the mean ± SEM.

Resident microglia and circulating peripheral monocytes can be distinguished by the expression of the chemokine receptor CX3CR1 because microglia highly express this receptor^43^,^44^. We thus used *Cx3cr1*^GFP/+^ mice as a model without irradiation chimerism or parabiosis surgery. Using flow cytometry, a single population of GFP^high^ cells (resident microglia) in the spinal cord in the contralateral side were detected (Fig. 4a). After PNI, the number of spinal GFP^high^ microglia increased (Fig. 4b), but the percentages of GFP^high^ and GFP^low or negative^ microglia in the ipsilateral spinal cord were almost identical to that in the contralateral side [GFP^high^: ipsilateral 99.7% and contralateral 99.6% (Fig. 4c); GFP^low or negative^: ipsilateral 0.3% and contralateral 0.4%]. Thus, these results suggest that circulating peripheral monocytes do not participate in the population of spinal microglia after PNI.

## Discussion

Microgliosis in the spinal cord induced by PNI has been assumed to be associated with a local expansion of resident microglia and/or with the recruitment of BM-derived cells from the bloodstream. Previous investigations in BM chimeric mice with irradiation at 10 Gy, a dose that is used for achieving full myeloablation^41^, showed infiltration of BM-derived cells into the spinal cord^31^,^32^,^33^,^34^. Consistent with these studies, we reproduced the spinal infiltration of GFP^+^ BM cells in irradiated mice (single dose of 10 Gy) and these GFP^+^ cells differentiated into microglia-like cells. While transplantation of BM cells to irradiated recipients (10 Gy) leads to high chimerism in the blood, it has been shown that such a high dose of whole-body irradiation induces inflammatory mediators and obvious BBB changes^38^,^39^,^40^,^41^. In this study, splitting irradiation using repeated lower doses, which results in milder toxicity than a single strong dose^41^, revealed that exposing mice to low doses of irradiation (5 Gy × 2) dramatically reduced the infiltration of CD11b^+^ BM-derived cells in the spinal cord compared with mice exposed to a single dose of 10 Gy, even though blood chimerism in both groups was almost identical (>97%). The marked reduction in spinal infiltration of CD11b^+^ GFP^+^ BM-derived cells was consistently observed in other groups with three different conditioning regimens. In these groups, the number of CD11b^+^ GFP^+^ cells was slightly increased in the spinal cord after PNI, which may be due to undesired side effects at lower doses^41^. Considering that all groups of BM chimeric mice induced PNI-induced spinal microgliosis, our findings suggest that the spinal recruitment of BM-derived cells after PNI, which has been reported previously, may be an experimental artefact. The artefact possibly results from irradiation that might cause BBB disruptions, and upregulation of chemokines and adhesion molecules, which leads to recruitment of cells^38^,^39^,^40^,^41^. To investigate the differential effect of the various radiation dosing regimens on blood-spinal cord-barrier (BSCB) permeability after PNI will require further investigation.

Our study using BM chimeric mice indicates few BM-derived CD11b^+^ GFP^+^ cells recruited to the spinal cord after PNI (although one limitation was to use the contralateral, but not sham-operated, spinal cord as controls). This is strongly supported by our further findings using the parabiosis model showing that in the absence of two experimental manipulations, irradiation and BM transplantation, there is no evidence of participation of BM cells to the population of spinal microglia after PNI. One technical limitation of parabiosis is the relatively lower frequency of blood chimerism than irradiated mice with BM transplantation. However, the lack of CD11b^+^ GFP^+^ cell recruitment seems to be selective to this cell type and to the spinal cord, as GFP^+^ cells in the DRG (which are presumably macrophages that are of haematogenous origin^47^,^48^) and CD11b^neg^ CD45^high^ GFP^+^ cells in the spinal cord were all detected after PNI. These data exclude the possibility that the little contribution of BM cells to spinal microglia populations is simply due to the level of circulating blood chimerism. From these results, we conclude that few BM-derived cells infiltrate into the spinal cord parenchyma after PNI and propose that microgliosis might be a consequence of a local expansion of spinal-resident microglia. This is supported by our data using *Cx3cr1*^GFP/+^ mice, a genetic approach without irradiation chimerism or parabiosis. Thus, our findings should prompt a careful reinterpretation of the previous literature describing the recruitment of BM-derived cells to the spinal cord after PNI and also using BM chimeric mice generated by a single high dose of whole-body irradiation.

Of course, all BM-derived cells do not necessarily infiltrate into the spinal cord after PNI. Indeed, we detected GFP^+^ cells with CD11b^neg^ CD45^high^, a profile that corresponds to lymphocytes including T cells. The PNI-induced recruitment of T cells in the spinal cord has been reported previously^49^,^50^,^51^. The mechanism for the infiltration of this cell-type after PNI remains unclear, but the PNI-induced expression of chemokines and dysfunction of BSCB may be involved^33^,^52^,^53^. Nevertheless, considering the limited number of CD11b^+^ BM-derived monocytes/macrophages in the ipsilateral spinal cord, such changes may not be sufficient for spinal infiltration of this cell type.

In the contralateral side of the spinal cord in irradiated mice (single dose of 10 Gy), the number of microglia was markedly decreased. The decrease appeared to occur in an irradiation dose-dependent manner, assuming the involvement of toxic inflammatory effects by the strong irradiation. Alternatively, given that resident microglia in the spinal cord are maintained by self-renewal^22^,^54^, the relatively high dose of irradiation may disrupt the proliferative capacity of resident microglia in the spinal cord^54^.

In conclusion, by using three different approaches, we provide evidence indicating little contribution of BM-derived cells in the population of spinal microglia after PNI. Thus, PNI-induced spinal microgliosis is not a consequence of infiltration of BM-derived monocytes/macrophages, but may rather be associated with a local expansion of resident microglia due to their proliferation activity in response to PNI^8^,^23^,^24^,^25^,^26^,^27^.

## Methods

### Animals

C57BL/6 (male and female), C57BL/6-Tg(CAG-EGFP) (male and female, Japan SLC) and B6.129P-*Cx3cr1*^*tm1Litt*^/J mice (male, Jackson Laboratory) were used. All mice were aged 4–10 weeks at the start of each experiment, and were housed in groups of two or three per cage at a temperature of 22 ± 1 °C with a 12-hour light-dark cycle, and were fed food and water *ad libitum*. All animal experiments were conducted according to the national and international guidelines contained in the ‘Act on Welfare and Management of Animals’ (Ministry of Environment of Japan) and ‘Regulation of Laboratory Animals’ (Kyushu University) and under the protocols approved by the Institutional Animal Care and Use committee review panels at Kyushu University.

### Generation of bone marrow chimera

Recipient C57BL/6 mice (male) at age 4–5 weeks were irradiated with various doses (1 dose of 5, 7 or 10 Gy; 2 doses of 3.5 or 5 Gy, 3-h interval) of γ-irradiation (Gammacell 40 Exactor) for ablation of endogenous BM cells. For BM transplantation, BM cells were isolated from adult CAG-EGFP mice (male, donor) by flushing the femurs and tibias using a 25G needle with Dulbecco’s modified Eagle medium (DMEM; Invitrogen). After resuspension, BM cells were centrifuged (300 × g, 5 min, 4 °C). After resuspension with ice-cold DMEM, BM cells were filtered through a 35-μm filter. Irradiated recipient C57BL/6 mice were anesthetised with isoflurane [2% (v/v)] and injected intravenously with 0.5–1.0 × 10^7^ donor BM cells (in 100 μL per recipient) through the retro-orbital sinus within 1 or 2 h after final irradiation. Four weeks after BM transplantation, a small amount of blood was collected through the retro-orbital sinus using a heparinised microhematocrit capillary (Fisher Scientific). The erythrocytes were lysed with a lysing buffer (BD Pharm Lyse^TM^, BD Bioscience), and after centrifugation (400 × g, 5 min, 4 °C), the leukocytes were washed in Hanks’ balanced salt solution (HBSS) including 2% (v/v) fetal bovine serum (FBS). Blood leukocytes were analysed by flow cytometry (FACSVerse) for GFP to examine the degree of chimerism of bone marrow cells. Three months after BM transplantation, BM chimeric mice were subjected to PNI.

### Peripheral nerve injury

We used the spinal nerve injury model with some modifications as described previously^55^. Under isoflurane [2% (v/v)] anaesthesia, a small incision at L3–S1 was made. Paraspinal muscle and fat were removed from the L5 traverse process, and the part of this traverse process was removed to expose the parallel-lying L3 and L4 spinal nerves, and then the L4 nerve was carefully isolated and cut. The wound and the surrounding skin were sutured with 5-0 silk and closed with staples.

### Immunohistochemistry

Mice were deeply anaesthetised by i.p. injection of pentobarbital and perfused transcardially with phosphate buffered saline (PBS), followed by ice-cold 4% (w/v) paraformaldehyde/PBS. The L4 segments of the spinal cord, or the L4 DRG were removed, postfixed in the same fixative for 3 h at 4 °C, and placed in 30% (w/v) sucrose solution for 24 h at 4 °C. Transverse L4 spinal cord sections (30 μm) and L4 DRG sections (15 μm) were incubated in blocking solution [3% (v/v) normal goat serum] for 2 h at room temperature and then incubated for 48 h at 4 °C with primary antibodies: rabbit polyclonal anti-Iba1 (1:5000, Wako), and rat monoclonal anti-CD11b (1:1000, Serotec), rat monoclonal anti-CD31 (1:200, BD Pharmingen) and hamster monoclonal anti-CD3 (1:100, eBioscience). Following incubation, tissue sections were washed and incubated for 3 h at room temperature in secondary antibody solution (Alexa Fluor 546 and Alexa Fluor 405, 1:1000, Molecular Probes, OR, USA). The tissue sections were washed, slide-mounted and subsequently coverslipped with Vectashield hardmount (Vector Laboratories). Three to five sections from the L4 spinal cord and DRG of each mouse were randomly selected and analysed using an LSM700 Imaging System (Carl Zeiss).

### Flow cytometry

Mice were deeply anaesthetised by i.p. injection of pentobarbital and perfused transcardially with PBS to remove circulating blood from the vasculature. The spinal cord was rapidly and carefully removed from the vertebral column and placed into ice-cold HBSS. The 3rd and 4th lumbar segments (2 mm long) of the spinal cord ipsilateral and contralateral to PNI were separated. Unilateral spinal tissue pieces were treated with pre-warmed 0.8-mL enzymatic solution [0.2 U/mL Collagenase D (Roche) and 4.3 U/mL of Dispase (GIBCO)] in HBSS-FBS for 30 min at 37 °C. The tissues were homogenised by passing through a 23G needle attached with a 1-mL syringe and were further incubated for 15 min at 37 °C. After that, the tissues were homogenised by passing twice through a 26G needle, and the enzymatic reaction was stopped by adding EDTA (0.5 M). Further processing was performed at 4 °C. After centrifugation (400 × g, 5 min, 4 °C), the pellets were resuspended in ice-cold HBSS-FBS. The resultant cell suspension [350 μL of the total suspension (500 μL)] was used for flow cytometry. The cell suspension was blocked by incubating with Fc Block (BD Pharmingen, 5 min, 4 °C) and immunostained with CD11b-A647 (BD Pharmingen, 1:10000, 30 min, 4 °C) and CD45-PerCP/Cy5.5 (BD Pharmingen, 1:1000, 30 min, 4 °C) in the dark. After washing, the pellet was resuspended in ice-cold HBSS-FBS and filtered through a 35-μm nylon cell strainer (BD Biosciences, San Jose, CA) to isolate tissue debris from the cell suspension. The total number of microglia in the L3/4 spinal cord was analysed using a FACSVerse flow cytometer (BD Bioscience) and FlowJo software (TreeStar).

### Parabiosis

Parabiotic pairs of female mice (CAG-EGFP and C57BL/6) were age-matched (6 to 7 weeks old) and housed together for 2 weeks before surgery. They were given a medicated Sulfa-Trim diet containing 0.124% (w/v) sulfamethoxazole and 0.025% (w/v) trimethoprim (TestDiet) 2 weeks prior to operation. Under isoflurane [2% (v/v)] anaesthesia, a mirror-image incision from elbow to knee along the flank of each mouse was made, and the subcutaneous fascia was bluntly dissected. Ligaments at the elbow and knee in each pair of mice were sutured together with 5-0 silk. The skin incisions were then closed with staples. Animals recovered in a heated cage. After the operation, each parabiont was subcutaneously injected with 1 mL of warm saline twice daily for 48 h. Two weeks after the operation, the medicated Sulfa-Trim diet was exchanged with a normal diet. One month after the operation, blood chimerism was analysed by flow cytometry. After that, parabionts (C57BL/6) were subjected to PNI and, 2 weeks later, the number of GFP^+^ cells in the spinal cord and DRG was analysed by flow cytometry and immunohistochemistry.

### Statistical analysis

Statistical significance of differences was determined using two-way ANOVA with post hoc Bonferroni test (Fig. 2b,c), one-way ANOVA with post hoc Dunnett’s multiple comparison test (Fig. 2b,c) or Paired Student’s t test (Figs 3b and 4b,c) using GraphPad Prism 4 software. Differences were considered significant at P < 0.05.

## Additional Information

**How to cite this article**: Tashima, R. *et al.* Bone marrow-derived cells in the population of spinal microglia after peripheral nerve injury. *Sci. Rep.* **6**, 23701; doi: 10.1038/srep23701 (2016).
